# Effect of Adding L-carnitine to High-Fat/Low-Protein Diets of Common Carp (*Cyprinus carpio*) and the Mechanism of Regulation of Fat and Protein Metabolism

**DOI:** 10.1155/2022/3768368

**Published:** 2022-08-23

**Authors:** Wei Luo, Pengyu Chen, Xiaoyang Zhang, Yibo Zhang, Shoudong Zhang, Kunpu Sun, Feifei He, Luojia Li, Ning Zhang, Yinlin Xiong, Zhonggang Guo, Zongjun Du, Anxiang Wen

**Affiliations:** ^1^Sichuan Agriculture University, College of Animal Science, Chengdu, China; ^2^Original Stock Farm Leiocassis Longirostris, Sichuan, Chengdu, China; ^3^Chongzhou Agriculture & Rural Bureau, Chengdu, China; ^4^Sichuan Agriculture University, College of Life Science, Yaan 625014, China

## Abstract

L-carnitine is a low molecular weight amino acid that plays an essential role in the oxidation of long-chain fatty acids. The regulatory effects and molecular mechanisms of L-carnitine on fat and protein metabolism in common carp (*Cyprinus carpio*) were investigated in this study. Common carp (*n* = 270) were randomly divided into three groups and fed either (1) common carp diet, (2) high-fat/low-protein diet, or (3) L-carnitine-high-fat/low-protein diet. Growth performance, plasma biochemistry, muscle composition, and ammonia excretion rate were all examined after 8 weeks. Additionally, each group's hepatopancreas was subjected to transcriptome analysis. The results showed that decreasing the feed protein/fat ratio resulted in a considerable increase in feed conversion ratio and a significant decrease in common carp-specific growth rate to 1.19 ± 0.02 (*P* < 0.05). Similarly, total plasma cholesterol sharply increased to 10.15 ± 2.07, while plasma urea nitrogen, muscle protein, and ammonia excretion levels dropped (*P* < 0.05). After adding L-carnitine to the high-fat/low-protein diet, it was found that the specific growth rate and protein content of the dorsal muscle increased significantly (*P* < 0.05). In contrast, the plasma total cholesterol and ammonia excretion rate decreased considerably at most time points after feeding (*P* < 0.05). The expression of genes in the hepatopancreas differed substantially between the different groups. Through GO analysis, it was demonstrated that L-carnitine increased the ability of fat decomposition by up-regulating the expression of *cpt1* in the hepatopancreas and decreased the expression of *fasn* and *elovl6* to reduce the production and extension of lipids. Simultaneously, *mtor* was more abundant in the hepatopancreas, implying that L-carnitine can increase protein synthesis. According to the findings, adding L-carnitine to high-fat/low-protein diets can stimulate growth by enhancing lipolysis and protein synthesis.

## 1. Introduction

The ability of fish to gain energy from carbohydrates is generally low, so it is necessary to add a high-protein content to the feed as an energy source [1]. However, high-quality protein costs have increased, and sources are limited. Therefore, in aquaculture, to reduce protein source energy consumption and nitrogen excretion, a reduced feed protein/fat ratio has been applied [2]. It is widely believed that increasing lipolytic function minimizes partial protein consumption [3]. A diet high in fat causes a significant accumulation of fat in the liver and abdominal regions, which leads to metabolic problems [4]. This causes further adverse reactions in the fish, including stunted growth and liver damage [5, 6]. Therefore, reducing or eliminating the adverse effects of high-fat/low-protein feed on fish via feed additives is crucial.

Carnitine (4-trimethylammonium-3-hydroxybutyric acid) is a quaternary ammonium compound in almost all animals, plants, and microorganisms. In animals, the primary sources of carnitine are exogenous intake and endogenous synthesis of lysine and methionine in the liver [7]. Carnitine is essential for the transport of long-chain fatty acids into mitochondria. There are two subtypes of carnitine: L-carnitine and D-carnitine [8]. It has been shown that D-type has almost no physiological value, while L-isomers have biological activity [9]. L-carnitine is mainly involved in energy metabolism and is the carrier of long-chain fatty acids entering mitochondria for *β*-oxidation. This can increase the oxidative energy supply of long-chain fatty acids [10], lowering total plasma cholesterol, boosting protein content, and decreasing body fat rate [11, 12].

There have only been a few studies that have explored the addition of L-carnitine to fish feed. For instance, supplementing goldfish (*Carassius auratus*) with 1000 mg kg^−1^ L-carnitine could dramatically boost growth performance and feed conversion rate [13]. Adding L-carnitine to Nile tilapia (*Oreochromis niloticus*) can effectively stimulate the lipid catabolism of muscle and increase the deposition of glycogen and protein [7]. In zebrafish (*Brachydanio rerio*), dietary L-carnitine can minimize lipid precipitation and enhance protein synthesis by improving mitochondrial fatty acid *β*-oxidation [10].

It is unclear what molecular mechanism L-carnitine uses to control metabolism in fish. Adding 500 mg kg^−1^ L-carnitine to a zebrafish diet containing 7% fat resulted in increased transcriptional activity of cpt1 (a gene involved in lipid breakdown) and decreased transcriptional activity of the lipid synthesis genes *acc*, *fasn*, and *dgat2* [10]. It has also been shown in largemouth bass (*Micropterus salmoides*) that L-carnitine could significantly upregulate the transcriptional level of the *cpt1* gene in the liver tissue [14]. In addition, it has been observed that dietary L-carnitine can positively influence the transcriptional expression of lipid synthesis-related genes in the liver of giant yellow croaker (*Pseudosciaena crocea*) [15].

Common carp is one of the most common farmed fish in Asia. In 2019, China alone produced 2,885,284 tons of common carp (China Fishery Statistical [16]). The effects of a high-fat/low-protein diet on growth and metabolism were investigated using common carp in this study. L-regulatory carnitine's effects on growth performance, plasma biochemistry, muscle composition, and ammonia excretion rate were also investigated. This was combined with the transcriptome sequencing technique to detect gene expression in the hepatopancreas. Therefore, this research is aimed at providing a theoretical basis for applying L-carnitine into feedstock for common carp aquaculture.

## 2. Materials and Methods

### 2.1. Experimental Fish and Diets

This study was carried out in the aquaculture experimental base's 9 outdoor cement ponds (length : width : height = 2 m × 2 m × 1.4 m) at the College of Animal Science and Technology, Sichuan Agricultural University. Common carp were purchased from the Sichuan Yuyuan farm. Healthy common carp (130 ± 6 g) (*n* = 270) were divided into three treatment groups with three replicates. For eight weeks, the fish were hand-fed at 9:00 and 18:00 every day, and the feeding volume was recorded (the feeding amount was 4-5% of the body weight of the fish). During the feeding period, the water temperature was kept at 27 ± 2°C, the pH at 7.4 ± 0.27, the dissolved oxygen level was not less than 6.0 mg/L, and the total ammonia level was less than 0.05 mg/L. Fish and soybean meal was used as a protein source and soybean oil as a fat source. Three different feeds were set up: Diet 1 (6% fat common carp primary feed), Diet 2 (protein reduction of 6% and fat increase of 4% based on control feed formula), and Diet 3 (adding 500 mg/kg L-carnitine based on Diet 2 recipe). L-carnitine is purchased from Cool Chemistry Co., Ltd. (Catalogue # PH362519), with purity > 98%. First, the feed required during the experiment is estimated to calculate the amount needed for various raw materials in each formula, according to Table 1.

Secondly, solid raw materials (premixes, flour, *α*-starch, dihydrogen phosphate, and L-carnitine) do not need to be crushed (they can be directly weighed). They are fed into the crusher (SFSP138 champion king crusher, Jiangsu) in Jiangsu Zhengchang Grain Machinery Co., Ltd. Dafa is gradually added to mix all raw materials. Subsequently, the feed mixture is generated using the laboratory feed granularity mechanism. The particles are air-dried at room temperature using an electric fan, with the room temperature saved for subsequent application.

### 2.2. Sampling and Processing

Daily feeding was recorded over 8 weeks, residual bait was weighed after air-drying, the number of fish deaths per day was recorded, and the survival rate was calculated. At the start of the experiment, the weight of the fish in each group was recorded. The final weight and total length of each group of fish were measured after a 24-hour fast, and the weight gain rate and specific growth rate were computed. Ten fish were randomly selected from each repeat, and blood was collected from the tail vein using a 1 mL syringe after anesthesia. The blood was placed into an EP (Eppendorf) tube, washed with 0.5% (*w*/*v*) sodium heparin, and centrifuged for 10 mins at 4°C. The upper plasma layer was extracted and frozen at -20°C. The gonads and hepatopancreas were then dissected and weighed, and each group's gonad and hepatopancreas index were calculated. The intestinal length was measured and weighed, and the intestinal length index and intestinal body index were calculated. An additional three fish were dissected, and their hepatopancreas was quickly frozen in liquid nitrogen and stored at -80°C for further analysis. The back muscles were frozen at -20°C for further muscle composition investigation. For determining growth performance, the following parameters were used:

(1) $$\begin{array}{c} \text{Survival rate }\left(\text{SR}\right)\%=\frac{\left(\text{Numbers of final alive fish}\right)}{\left(\text{Numbers of initial alive fish}\right)}\times 100,\\ \text{Weight gain rate }\left(\text{WGR}\right)\%=\frac{\left(\text{Final body weight}-\text{initial body weight}\right)}{\left(\text{Initial body weight}\right)}\times 100,\\ \text{Feed conversion ratio }\left(\text{FCR}\right)=\frac{\left(\text{Total feed on daily basis}\right)}{\left(\text{Weight gain}\right)}\times 100,\\ \text{Hepatopancreas index }\left(\text{HSI}\right)\%=\frac{\left(\text{Liver pancreas weight}\right)}{\left(\text{Whole body weight}\right)}\times 100,\\ \text{Gonad index }\left(\text{GI}\right)\%=\frac{\left(\text{Gonad weight}\right)}{\left(\text{Whole body weight}\right)}\times 100,\\ \text{Intestinal lenght index }\left(\text{ILI}\right)\%=\frac{\left(\text{Intestinal lenght}\right)}{\left(\text{Whole body lenght}\right)}\times 100,\\ \text{Intestinosomatic index }\left(\text{ISI}\right)\%=\frac{\left(\text{Intestinal weight}\right)}{\left(\text{Whole body weight}\right)}\times 100,\\ \text{Specific growth rate }\left(\text{SGR}\right)\%=\frac{\left(\text{In final weight}\right)-\left(\text{In initial weight}\right)}{\left(\text{Days}\right)}\times 100. \end{array}$$

### 2.3. Ammonia Nitrogen Excretion

The remaining fish were placed in glass aquariums with numbers corresponding to the original group. The fish fasted for two days after a week of temporary culture (the fish were fed the same diet as the culture study). The fish tank was thoroughly cleaned on the third day, and each group of fish was weighed. The fish were then fed again, the residual bait collected after eating, and a 100 mL water sample was taken from each tank. Nessler's colorimetric method was used to determine the ammonia nitrogen concentration in the water samples. Subsequently, 100 mL water samples were taken every hour to determine the concentration of ammonia nitrogen *C*_*n*_ (*n* = 1, 2, 3, 4, 8). The average ammonia excretion rate is calculated using the following formula:

(2) $$\begin{array}{c} \text{Average ammonia excretion rate}=\frac{\left(C_{n}-C_{0}\right)}{\left(n\;x\;M\right)}, \end{array}$$

where *C*_0_ represents the ammonia nitrogen concentration of the first water sample, *C*_*n*_ denotes the ammonia nitrogen concentration of the water sample after being fed with *n* *h*, and *M* represents the body weight of the fish.

### 2.4. Biochemical Parameters Measurements

Plasma concentrations of triacylglycerol, total cholesterol, and urea nitrogen were measured using commercial kits (Jiancheng Biotech Co., Ltd., Nanjing province, China). The crude protein content of the back muscle was determined using the Kjeldahl method (Nx6.25), and the crude fat content was extracted using the Soxhlet procedure.

### 2.5. Transcriptome Assay

Three fish were collected from each of the three groups to investigate the different diet groups' differential gene expression in the hepatopancreas. After dissecting the hepatopancreas, total RNA was extracted using a “Total RNA extraction kit” (TaKaRa Biotechnology (Dalian) Co., Ltd., Dalian, China). An Agilent 2100 BioAnalyzer® (Agilent Technologies, Palo Alto, CA, USA) was used to confirm the integrity of the RNA and build the cDNA library. The samples were prepared with an Illumina kit (Illumina, Inc., San Diego, CA, USA). An Agilent 2100 Bioanalyzer was then used to check the quality of the library. The common carp hepatopancreas cDNA library was sequenced by a double terminal (paired-end (PE)) using an Illumina HiSeqTM2000 platform. “Cutadapt” was used to remove reads with joints and reads with an average mass fraction lower than Q20. The filtered reads were compared with the reference gene (GCF_000951615.1) by HISAT2 software. FPKM (Fragments Per Kilo bases per Million fragments) was used to standardize the expression (normalization). The criteria for screening differentially expressed genes were multiple differential expression |*log*2*FoldChange*| > 1 and *P* < 0.05, using DESeq to evaluate gene expression variation. The findings demonstrated that the level of differentially expressed genes was modified. The GO database was examined for known pathways in which DEGs were enhanced. KEgg pathway analysis was also conducted on DEGs to discover pathways with highly enriched content.

### 2.6. Statistical Analysis

Statistical analysis was performed using SPSS20.0 software (SPSS, Chicago, IL, USA). All results are presented as the mean ± SEM. Following confirmation of the data's normality and variance homogeneity, significant differences were discovered using one-way analysis of variance (ANOVA), accompanied by Duncan's multiple range test. A difference of *P* < 0.05 was considered significant.

### 2.7. Gene Expression Validation

To verify the RNA-Seq data, the transcriptome RNA samples were quantified using quantitative real-time PCR (qRT-PCR). Four protein and lipid metabolism-related genes were chosen: elongation of long-chain fatty acid family member 6 (*elovl6*), lanosterol synthase (*lss*), mammalian target of rapamycin (*mtor*), and glycine amidinotransferase (glycine amidinotransferase) (*gatm*). Primers were designed using Primer Premier 5 software (Table S1). qRT-PCR with SYBR Green dye (Takara, Japan) was performed on an ABI PRISM 7500 Fast Real-Time PCR System according to the manufacturer's instructions. All reactions were carried out in triplicate. The target specificity was determined through melting curve analysis. The 22^-*ΔΔ*CT^ method was applied to generate the qRT-PCR data, with *β*-actin as the internal control. All samples were examined in triplicate.

## 3. Results

### 3.1. Growth and Feeding Parameters

Table 2 depicts the common carp's growth performance. Diet 1 had a substantially higher weight gain rate (WGR) (185.22 ± 29.82%) than Diet 2 and Diet 3 (*P* < 0.05). Diet 2 had a significantly greater feed conversion ratio (FCR) (1.68 ± 0.17) than Diet 1, whereas Diet 3 was in the middle and revealed no significant differences. The specific growth rate (SGR) of Diet 1 (1.53 ± 0.04) was significantly higher than that of Diet 2, while Diet 3 was between the two and showed a statistical difference from the other two (*P* < 0.05). The intestinal length index (ILI) and intestinosomatic index (ISI) of Diet 1 were not significantly different from those of Diet 2 but were considerably lower than those of Diet 3 (*P* < 0.05). The three groups did not significantly differ in gonad index (GI) or hepatopancreas index (HSI).

### 3.2. Proximate Composition and Blood Parameters

The total plasma cholesterol (TCHO) of Diet 2 was 10.15 ± 2.07 mmol/L, and the plasma urea nitrogen (BUN) of Diet 1 was 0.83 ± 0.13 (Table 3), which were both significantly higher than that of the other two groups (*P* < 0.05). Diet 1 had the most protein in the dorsal muscle, followed by Diet 3, and Diet 2 had the least; there was a statistically significant difference between the three groups (*P* < 0.05). There was no substantial difference between the 3 groups in triglyceride (TG) levels (0.74 ± 0.28 to 0.89 ± 0.30 mmol/L) or fat content in the dorsal muscle (2.29 ± 0.22 to 2.73 ± 0.61%, *P* > 0.05).

### 3.3. Ammonia Excretion Rate

**Figure 1:**
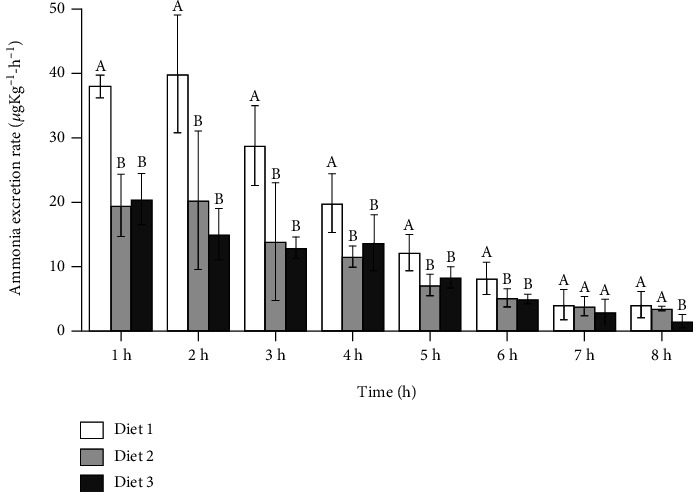
Change in ammonia excretion rate.

Figure 1 depicts the change in ammonia excretion rate in the three groups 1-8 h after feeding. In Diet 1 and Diet 2, ammonia excretion rates increased 2 h after feeding, while in Diet 3, the ammonia excretion rate maximum of 1 h after feeding. The three groups' ammonia excretion rates exhibited slight variation and tended to stay regular 7-8 h after the meal. The ammonia excretion rate of the three groups showed a downward trend after the peak. In general, the ammonia excretion rate of Diet 1 was the highest, followed by Diet 2 and Diet 3, and Diet 2 was higher than Diet 3 at most time points.

### 3.4. Transcriptome Sequencing Analysis

#### 3.4.1. The Quality of Library Sequencing and DEG Analysis

**Figure 2:**
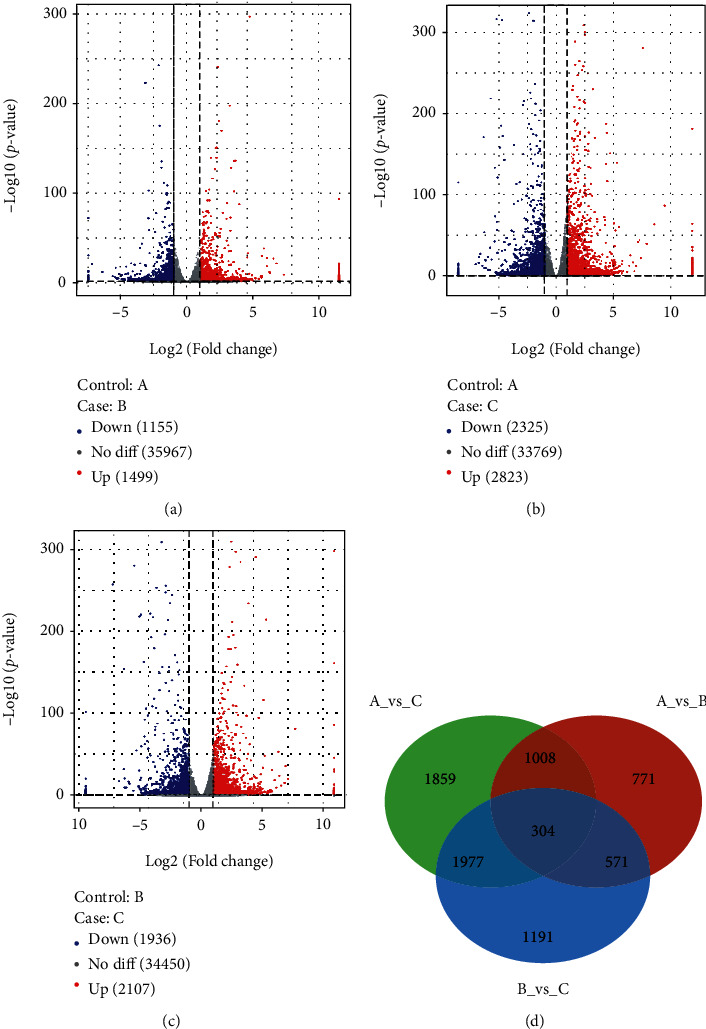
Volcano map of differentially expressed genes. Note: case, experimental group sample; control, comparative group sample. In the figure, the two vertical dashes are 2 times the expression difference threshold, and the horizontal dashes are *P* value = 0.05 threshold. The red dot indicates the upregulated gene, the blue dot indicates the downregulated gene, and the grey dot indicates the gene with no significant difference. (a) The upregulated or downregulated genes in the Diet 2 were compared with those in Diet 1. (b) The upregulated or downregulated genes in the Diet 3 were compared with those in Diet 1. (c) The upregulated or downregulated genes in the Diet 3 were compared with those in Diet 2. (d) Comparison among three groups, resulting in gene difference.

A total of 380,197,480 original sequences (125,495,130 in Diet 1, 119,484,898 in Diet 2, and 135,217,452 in Diet 3) were obtained, including 353,938,530 clean reads (116,812,418 in Diet 1, 111,200,472 in Diet 2, and 125,925,640 in Diet 3) (Table S2). A percentage of Q30 higher than 93.30% was obtained for follow-up analysis. After mapping the annotated common carp genome, 278,226,421 sequences were successfully located. Comparative analysis of changes to the liver transcriptome was performed to identify DEGs. Diet 2 has 2,654 genes differentially expressed compared to Diet 1, 1,499 of which were up-regulated (Figure 2). Compared to Diet 1, 2823 genes were upregulated, and 2325 were downregulated in Diet 3. Diet 3 upregulated 2107 genes and downregulated 1936 genes compared to Diet 2. There were 304 DEGs found, with substantial variations in the hepatopancreas amongst the three groups.

#### 3.4.2. KEGG Enrichment Analysis

KEGG analyzed the DEGs in the hepatopancreas of the three groups. The DEGs between Diet 1 and Diet 2 were enriched in 146 pathways. The enrichment of KEGG of genes with higher or lower expression of Diet 2 in the hepatopancreas was examined using Diet 1 as a standard. The 20 pathways with the highest significant enrichment were picked for representation (Table S3). It was found that the two groups' metabolic pathways of DEG enrichment were mainly related to fat metabolism, amino acid-like metabolism, exogenous substance degradation metabolism, and cell growth and metabolism. In the KEGG enrichment table, the KEGG classifications linked to fat and amino acid metabolism were discovered, and DEGs performing these biological functions were screened (Table S4).

The DEGs between Diet 2 and Diet 3 were enriched in 158 pathways. Taking Diet 2 as a reference, the enrichment of KEGG of genes with increased or decreased expression in the hepatopancreas of Diet 3 was counted, and the 20 pathways with the most significant enrichment were selected for display (Table S5). The two groups' metabolic pathways of DEG enrichment were found to be mainly related to fat metabolism, carbohydrate metabolism, cell growth and death, amino acid metabolism, and exogenous biodegradation metabolism. In the KEGG enrichment table, the KEGG classifications associated with fat and amino acid metabolism were discovered, and DEGs executing these biological functions were examined (Table S6).

#### 3.4.3. GO Enrichment Analysis

**Figure 3:**
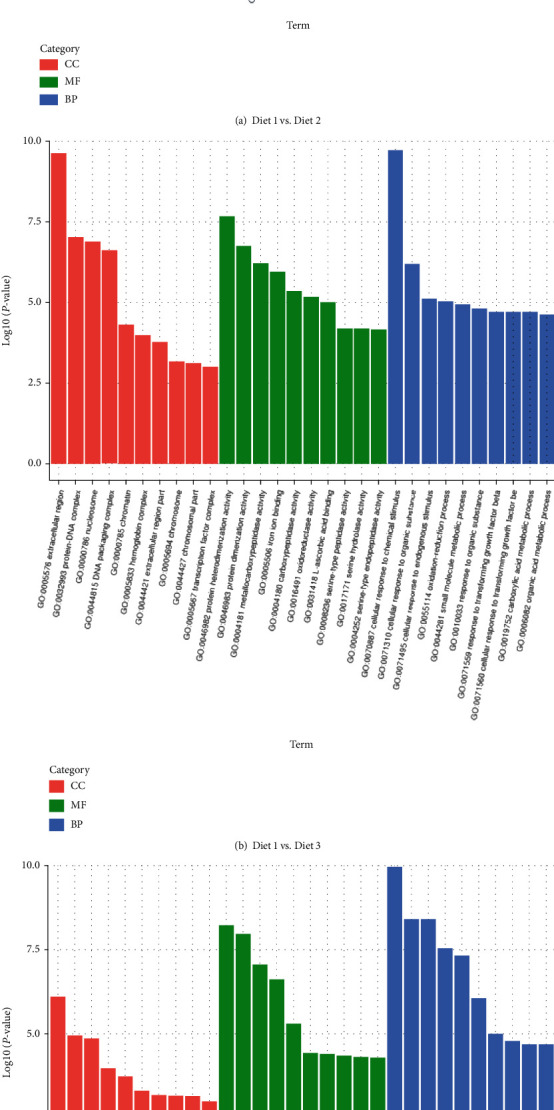
Histogram of GO enrichment analysis. The *x*-coordinate is the term of go Level 2; and the *y*-coordinate is -log_10_ (*P* value) enriched by each term.

The number of GO items examined between Diet 1 and Diet 2 was the least, and the differentially expressed genes between the three groups were mainly enriched in biological processes, according to GO analysis of DEGs in the hepatopancreas of the 3 groups of fish (Table 4). For the exhibition, the first 20 GO items with substantial enrichment between the two groups were considered (Figure 3).

The molecular functions and biological processes related to protein and DNA synthesis between Diet 1 and Diet 2 accounted for the highest proportion, mainly including “peptidase activity,” “serine-type peptidase activity,” “serine hydrolase activity,” “serine-type endopeptidase activity,” “peptidase activity, acting on L-amino acid peptides,” “proteolysis,” “cell redox homeostasis,” “metabolic process,” “DNA modification,” and “DNA alkylation.”

Diet 2 and Diet 3 accounted for the highest proportion of growth factors, redox related biological processes and molecular functions, including “small molecule metabolic process,” “organic acid metabolic process,” “oxoacid metabolic process,” “carboxylic acid metabolic process,” “oxidation-reduction process” “iron ion binding” “tetrapyrrole binding,” “oxidoreductase activity,” “heme binding,” and “vitamin binding.”

#### 3.4.4. Gene Annotation Analysis

Diet 2 increased the expression of *elovl6* (a gene related to fatty acid synthesis) and *pik3r3* (a gene associated with fatty acid catabolism) while decreasing the expression of lipc (a key enzyme in lipoprotein metabolism), *lars*, *gss*, *mtor*, *gch1*, *zmpste24*, *tmem27*, *pm20d1*, and other protein metabolism genes. Compared with Diet 2, Diet 3 downregulated the fat synthesis-related gene *elovl6* and upregulated the expression of *cpt1*, c*pt2*, and other genes related to fatty acid decomposition, among which the *cpt2* gene was involved in the regulation of fatty acid-*β*-oxidation. The expression of the protein metabolism-related genes *wars*, *cpb1*, and *cela1* was downregulated, while the expression of *zmpste24*, *mtor*, and *gatm* was upregulated (Table S7).

### 3.5. Gene Expression Validation

**Figure 4:**
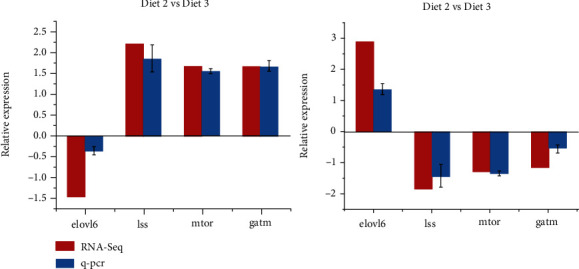
Validation by quantitative RT-PCR of four differentially expressed genes identified by RNA-Seq in the hepatopancreas of common carp.

To confirm the DEG findings, two genes involved in protein metabolism and two in fat metabolism were determined for qRT-PCR investigation. As shown in Figure 4, the results demonstrated that the expression levels of the genes studied were comparable with the RNA-Seq results. These findings validated the trustworthiness of the RNA-Seq findings.

## 4. Discussion

### 4.1. The Effect of a High-Fat/Low-Protein Diet

This study found that the WGR and SGR of common carp were significantly decreased, and the feed conversion ratio was significantly increased after feeding a high-fat/ow-protein diet for 8 weeks. The growth performance of largemouth fish on a high-fat diet followed the same pattern [17]. In addition, studies have shown that Atlantic salmon (*Salmo Salar*) fed high-fat/low-protein diets have lower energy utilization, resulting in slower growth [18]. These results indicate that the diet's low protein/fat ratio significantly affected the development of common carp.

The increase in TCHO content in plasma will improve blood viscosity, resulting in hyperlipidemia, which is not conducive to the healthy growth of fish. The plasma TCHO of carp was substantially enhanced after feeding with a high-fat/low-protein diet in this experiment, putting the common carp's health at risk; similar results have been found with large yellow croaker [6], blunt snout bream (*Megalobrama amblycephala*) [19], and rice field eel (*Monopterus albus*) [20]. Ammonia is the end product of amino acid catabolism, accounting for 80-90% of the body's total nitrogen excretion [21]. BUN level has been used to assess urinary ammonia excretion in animals, and its change can reflect differences in protein catabolism levels and nitrogen excretion [2]. Diet 2 had a much lower BUN concentration and ammonia excretion rate than Diet 1, indicating that the protein breakdown level is lower, and energy is obtained by reducing protein decomposition, thereby preserving feed protein.

Furthermore, the ammonia excretion rate of Diet 1 was much higher than that of the other two groups, indicating a positive correlation between ammonia excretion and dietary protein content. Similar results were obtained in the bass (*Dicentrarchus labrax*) [22] and flounder (*Paralichthys olivaceus*) [23]. Diet 2 had much less protein deposition than Diet 1, implying that a reduction in protein intake from the diet would lead to a decrease in protein deposition in carp muscle. In gibel carp (Carassius gibelio), similar results were observed [24] and rice field eel [20].

Diet 1 and 2 hepatopancreas transcriptome expressions were compared. Diet 2 was observed to suppress the expression of *lars*, *gss*, *zmpste24*, *gch1*, *tmem27*, *pm20d1*, and *mtor* compared to Diet 1. The expression of lipid metabolism-related genes *pik3r3* and *elovl6* was upregulated. *Cpt1* and *cpt2* are critical genes related to lipid decomposition [25], but they have not been screened in the enriched pathways. These findings suggest that when fat consumption increased, the ability of common carp to decompose lipids did not rise considerably. Despite this, the capacity for protein metabolism was reduced after consuming high-fat/low-protein diets.

### 4.2. The Regulation of L-carnitine on Fat Metabolism

The SGR of Diet 3 was significantly higher than that of Diet 2, indicating that adding 500 mg/kg·L^−1^ L-carnitine in the high-fat/low-protein diet could substantially improve the growth performance of common carp. The same trend was found in studies of large yellow croaker [26], golden pompano (*Trachinotus ovatus*) [27], and juvenile derbio (*Trachinotus ovatus*) [28]. It was also found that after the addition of L-carnitine, the dorsal muscle showed a downward trend of fat deposition. Similarly, in studies of Atlantic salmon [29], silver perch (*Bidyanus bidyanus*) [30], and zebrafish [10], dietary L-carnitine reduced muscle fat. In this investigation, the plasma TCHO level of Diet 3 was much lower than that of Diet 2, with no statistical difference between Diets 3 and 1, showing that L-carnitine can reduce the harmful effect of excess fat in diets on the hepatopancreas by lowering plasma TCHO levels. This is congruent with the findings in rainbow trout (*Oncorhynchus mykiss*) [11] and silver perch [30].

Diet 2 and Diet 3 differentially expressed genes associated with fat metabolism were investigated. Data from sequencing revealed that 19 DEGs were strongly associated with the “steroid biosynthesis” signaling pathway inside the fat metabolic system. In addition, it was found that compared with Diet 2, Diet 3 downregulated the elovl6 and fasn and upregulated the *cpt1* and *cpt2*. *Elovl6* is a member of the ultra-long-chain fatty acid elongation enzyme (*ELOVLs*) family and is involved in fatty acid elongation and biosynthesis of lipoyl CoA, which regulates the synthesis of ultra-long-chain fatty acid elongation enzymes. *Elovl6* has been extensively studied in mammals, but little is known about its role in fish nutritional regulation [31]. *Cpt1a* (carnitine O-palmityl transferase 1, liver subtype) is a type of *cpt1*, which is mainly expressed in hepatopancreas and forms the carnitine transport system together with *cpt2* (carnitine O-palmityl transferase 2) and acylcarnitine carnitine transporter to participate in fatty acid decomposition [10]. The upregulation of *cpt1a* and *cpt2* expression suggested that L-carnitine could enhance the body's lipid oxidation capability, similar to recent findings in zebrafish [10] and largemouth bass [14]. *Fasn* can regulate the amount of fatty acid synthase, which determines the speed and direction of reaction in the *de novo* synthesis pathway of long-chain fatty acids [32]. Previous research has demonstrated that high fatty acid synthase activity can promote the conversion of malondialdehyde CoA into fatty acids, which can then be esterified to produce fat. Studies in poultry and mammals have revealed that fasn expression is directly correlated with animal body fat levels and can be used as a genetic marker for lipid metabolism [15]. The discrepancy in the expression levels of these genes shows that L-carnitine regulates the lipid metabolism of common carp by upregulating the expression levels of *cpt1* and *cpt2* to improve the lipid decomposition ability and downregulating the expression levels of *fasn* and *elovl6* to affect lipid generation and extension, thus reducing the accumulation of lipid. The same pattern was discovered in young Nile tilapia research [33].

### 4.3. The Regulation of L-carnitine on Protein Metabolism

Compared with Diet 2, the protein content of dorsal carp muscle was significantly increased in Diet 3. It is suggested that adding L-carnitine in high-fat/low-protein diets can increase the protein content in the body of common carp. The fundamental explanation could be that higher external L-carnitine intake leads to feedback inhibition of critical enzymes involved in carnitine synthesis, which inhibits L-carnitine formation by the body from lysine and methionine, so raises protein level in the body [10]. It is also likely that L-carnitine alters the direction of waste nitrogen transformation in metabolism by enhancing pyruvate carboxylase and lowering branch chain *α*-ketoate dehydrogenase complex, hence promoting protein synthesis in the body [34]. There was no statistical change in BUN between Diet 2 and Diet 3, demonstrating that BUN in plasma was predominantly positively connected with dietary protein intake, with no strong correlation with whether L-carnitine was supplemented to the diet. According to previous research on largemouth bass [35], *Pampus argenteus* (Euphrasen) [36], and tiger puffer (*Takifugu rubripes*), L-carnitine does not affect protein deposition in muscle by changing the content of BUN in plasma during the regulation of protein metabolism in fish [37]. It was also found that the ammonia excretion rates of the three groups of common carp all had a downwards trend 3 hours after reaching the peak value, indicating that the fish had a certain ammonia nitrogen excretion rhythm after feeding. This was consistent with the studies on mandarin fish (*Siniperca chuatsi*) [21] and grass carp (*Ctenopharyngodon idellus*) [21]. At most times, the ammonia excretion rate of Diet 3 was lower than that of Diet 2, presumably because L-carnitine improved the oxidation of dietary fat to meet the body's energy requirements, hence decreasing protein intake and increasing nitrogen reserve [10].

The DEGs associated with protein metabolism that differed across Diets 2 and 3 were subjected to further screening. Twenty DEGs were strongly linked to arginine and proline metabolism signaling pathways within the amino acid metabolism system, as revealed by our sequencing data. In addition, analysis of DEGs identified in Diets 2 and 3 showed that L-carnitine downregulated the gene expression levels of *acmsd*, *wars*, *gpr143*, *cpb1*, *cela1*, and *slc6a8*, while genes such as *zmpste24*, *mtor*, and *gatm* were upregulated. Among them, *zmpste24* [38] and *cela1* [39] is involved in protein decomposition, while *mtor* is a crucial regulator of protein metabolism (Ball et al., 2017). According to research, the *mTOR* protein generated after *mTOR* expression is the core of the *mTOR* pathway, which, along with Raptor, *PRAS40*, Deptor, *mLST8*, *Tel2*, and *Ttil*, forms the mTORC1 complex protein and is regulated by the PI3K/PKB (protein kinase B) signaling pathway, which can sense various nutritional factors, growth factors (IGF-1, IGF-2) ([40]; Ball et al., 2017). This can subsequently impact the activity of downstream signal molecules or mRNA translation, regulating protein synthesis and participating in various physiological and biochemical activities [41]. The molecular mechanism can explain this; although the plasma BUN content in Diet 3 group was not significantly different from that in Diet 2, the protein content in muscle was still increased.

## 5. Conclusion

The growth of carp was significantly affected by a high-fat/low-protein diet. Adding 500 mg/kg L^−1^ L-carnitine to the high-fat/low-protein diet greatly improved protein and fat metabolism. Adding L-carnitine to high-fat/low-protein diets can promote the high expression of fat oxidization-related genes *cpt1a* and *cpt2* and the low expression of fat synthesis-related genes *fasn* and *elovl6*. At the same time, the protein metabolism of the common carp is regulated by the high expression of *mtor*, the core protein of the mTOR pathway.

## Figures and Tables

**Table 1:** Composition and proximate analysis of experimental diets.

Ingredients (g/kg)	Diet 1	Diet 2	Diet 3
Fishmeal	280	280	280
Soybean meal	300	150	150
Flour	100	150	150
Wheat bran	60	80	80
Soybean oil	20	60	60
Vegetable meal	40	20	20
Corn gluten	100	160	160
Dihydrogen phosphate	10	10	10
Premix^1^	10	10	10
Alpha starch	80	80	79.5
L-carnitine	/	/	0.5
Total	1000	1000	1000
Proximate composition (% of dry matter)			
Protein	36.39	30.66	30.65
Ash	6.87	6.12	6.12
Lipids	5.93	9.97	9.97

^1^This premix in per kg diet support compositions: VA 400 000 IU; VD 50 000 IU; VC 750 mg; VE 200 mg; VB_1_ 15 mg; VB_2_ 75 mg; VB_6_ 22 mg; VK_3_ 65 mg; niacin 76 mg; calcium pantothenate 350 mg; biotin 550 mg; inositol 100 mg; Fe 156 mg; Cu 2.6 mg; Zn 70 mg; Mn 17 mg; Mg 300 mg; Co 0.2 mg; I 0.25 mg; Se 0.3 mg.

**Table 2:** The effect of L-carnitine on growth of common carp.

Item	Diet 1	Diet 2	Diet 3
Survival rate (%) (SR)	100^a^	100^a^	100^a^
Initial body weight (g) (IBW)	122.50 ± 26.29^a^	126.33 ± 24.65^a^	127.50 ± 19.89^a^
Final body weight (g) (FBW)	349.11 ± 36.50^a^	287.33 ± 36.58^b^	334.67 ± 47.43^a^
Weight gain rate (%) (WGR)	185.22 ± 29.82^a^	127.50 ± 28.96^b^	151.94 ± 38.74^b^
Feed conversion ratio (%) (FCR)	1.26 ± 0.21^b^	1.68 ± 0.17^a^	1.38 ± 0.25^ab^
Specific growth rate (%) (SGR)	1.53 ± 0.04^a^	1.19 ± 0.02^c^	1.30 ± 0.04^b^
Intestinal length index (%) (ILI)	129.14 ± 33.34^b^	128.19 ± 11.69^b^	160.29 ± 11.77^a^
Intestinal weight index (%) (ISI)	2.10 ± 0.32^b^	2.40 ± 0.52^ab^	2.75 ± 0.24^a^
Gonad index (%) (GI)	1.48 ± 1.02^a^	1.40 ± 0.59^a^	2.12 ± 1.06^a^
Hepatopancreas index (%) (HSI)	1.99 ± 0.24^a^	1.70 ± 0.32^a^	1.94 ± 0.38^a^

Different small letters in the same line represent a significant difference (*P* < 0.05), the same below.

**Table 3:** Effects of L-carnitine on serum biochemical and muscle composition of common carp.

	Diet 1	Diet 2	Diet 3
TCHO (mmol/L)	4.77 ± 1.15^b^	10.15 ± 2.07^a^	5.97 ± 1.32^b^
TG (mmol/L)	0.88 ± 0.54^a^	0.89 ± 0.30^a^	0.74 ± 0.28^a^
BUN (mmol/L)	0.83 ± 0.13^a^	0.65 ± 0.14^b^	0.61 ± 0.04^b^
Protein content (%)	16.65 ± 0.07^a^	15.93 ± 0.09^c^	16.37 ± 0.08^b^
Fat content (%)	2.73 ± 0.61^a^	2.34 ± 0.21^a^	2.29 ± 0.22^a^

**Table 4:** GO analysis of differentially expressed genes in hepatopancreas transcriptome.

	Toll GO	BP	CC	MF
A vs. B	3958	2634	413	917
A vs. C	4810	3176	535	1099
B vs. C	4782	3134	552	1126

In this study, the DEGs were classified into three categories: BP: biological process; MF: molecular function; CC: cellular component.

## Data Availability

The data used to support the findings of this study are available from the corresponding author upon request.

## Supplementary Materials

Supplementary 1 Supplementary Table 1: real-time PCR primer sequences.

Supplementary 2 Supplementary Table 2: clean data obtained from RNA sequencing.

Supplementary 3 Supplementary Table 3: top 20 KGEE items with significant enrichment in Diet 1 vs. Diet 2.

Supplementary 4 Supplementary Table 4: differential genes related to fat metabolism or protein metabolism in Diet 1 vs. Diet 2.

Supplementary 5 Supplementary Table 5: top 20 KEGG items with significant enrichment in Diet 2 vs. Diet 3.

Supplementary 6 Supplementary Table 6: differential genes related to fat metabolism or protein metabolism in Diet 2 vs. Diet 3.

Supplementary 7 Supplementary Table 7: differentially expressed genes related to fat or protein metabolism.

## References

[1] Wilson R. P. (1994). Utilization of dietary carbohydrate by fish. *Aquaculture*.

[2] Wang J. T., Han T., Li X. Y. (2017). Effects of dietary protein and lipid levels with different protein-to-energy ratios on growth performance, feed utilization and body composition of juvenile red-spotted *grouper,Epinephelus akaara*. *Aquaculture Nutrition*.

[3] Boujard T., Gélineau A., Covès D. (2004). Regulation of feed intake, growth, nutrient and energy utilisation in European sea bass (*Dicentrarchus labrax*) fed high fat diets. *Aquaculture*.

[4] Chaitanawisuti N., Kritsanapuntu S., Santaweesuk W. (2011). Effects of dietary protein and lipid levels and protein to energy ratios on growth performance and feed utilization of hatchery-reared juvenile spotted Babylon (*Babylonia areolata*). *Aquaculture International*.

[5] He A. Y., Ning L. J., Chen L. Q. (2015). Systemic adaptation of lipid metabolism in response to low- and high-fat diet in Nile tilapia (*Oreochromis niloticus*). *Physiological Reports*.

[6] Wang X. X., Li Y. J., Hou C. L., Gao Y., Wang Y. Z. (2015). Physiological and molecular changes in large yellow croaker (*Pseudosciaena* croceaR.) with high-fat diet-induced fatty liver disease. *Aquaculture Research*.

[7] Li L. Y., Lu D. L., Jiang Z. Y. (2020). Dietary L-carnitine improves glycogen and protein accumulation in Nile tilapia via increasing lipid-sourced energy supply: an isotope-based metabolic tracking. *Aquaculture Reports*.

[8] Vernez L. (2005). *Analysis of carnitine and acylcaritines in biological fluids and application to a clinical study*.

[9] Li J. M., Li L. Y., Zhang Y. X. (2019). Functional differences between l- and d-carnitine in metabolic regulation evaluated using a low-carnitine Nile tilapia model. *The British Journal of Nutrition*.

[10] Li J. M., Li L. Y., Qin X. (2017). Systemic regulation of L-carnitine in nutritional metabolism in zebrafish, Danio rerio. *Scientific reports*.

[11] Dikel S., Unalan B., Eroldogan O. T., Hunt A. O. (2010). Effects of dietary L-carnitine supple-mentation on growth, muscle fatty acid composition and economic profit of rainbow trout (*Oncorhynchus mykiss*). *Turkish Journal of Fisheries and Aquatic Sciences*.

[12] Ozorio R. O. A., Van Ginneken V. J. T., Verstegen M. W. A., Verreth J. A. J., Huisman E. A. (2010). Effects of exercise on L-carnitine and lipid metabolism in African catfish (*Clarias gariepinus*) fed different dietary L-carnitine and lipid levels. *The British Journal of Nutrition*.

[13] Arslan M. N., Ozbas M. (2020). Determination of growth performance and feed utilization of fry of goldfish, *Carassius auratus*(actinopterygII:cypriniformes: Cyprinidae) fed L-carnitine-supplemented diets. *Acta Ichthyologica et Piscatoria*.

[14] Chen Y., Sun Z., Liang Z. (2020). Addition of l-carnitine to formulated feed improved growth performance, antioxidant status and lipid metabolism of juvenile largemouth bass, *Micropterus salmoides*. *Aquaculture*.

[15] Zheng J. L., Luo Z., Zhuo M. Q. (2014). Dietary L-carnitine supplementation increases lipid deposition in the liver and muscle of yellow catfish (*Pelteobagrus fulvidraco*) through changes in lipid metabolism. *British Journal of Nutrition*.

[16] Yearbook C. F. S. (2020). *Fishery Bureau. Ministry of Agriculture*.

[17] Yin P., Xie S. W., Zhuang Z. X. (2021). Dietary supplementation of bile acid attenuate adverse effects of high-fat diet on growth performance, antioxidant ability, lipid accumulation and intestinal health in juvenile largemouth bass (*Micropterus salmoides*). *Aquaculture*.

[18] Weihe R., Rorvik K. A., Thomassen M. S., Asche F. (2019). A model system to evaluate the economic performance of two different dietary feeding strategies in farmed Atlantic salmon (*Salmo salar* L.). *Aquaculture*.

[19] Lu K. L., Xu W. N., Li J. Y., Li X. F., Huang G. Q., Liu W. B. (2013). Alterations of liver histology and blood biochemistry in blunt snout bream *Megalobrama amblycephala* fed high-fat diets. *Fisheries Science*.

[20] Yue H. M., Huang X. Q., Ruan R., Ye H., Li Z., Li C. J. (2020). Effects of dietary protein levels on the growth, body composition, serum biochemistry and digestive enzyme activity in Chinese rice field eel (*Monopterus albus*) fingerlings. *Aquaculture Research*.

[21] Fang L., Bai X. L., Liang X. F. (2017). Ammonia nitrogen excretion in mandarin fish (*Siniperca chuatsi*) and grass carp (*Ctenopharyngodon idellus*) fed practical diets: the effects of water temperature. *Aquaculture Research*.

[22] Peres H., Oliva-Teles A. (2002). Utilization of raw and gelatinized starch by European sea bass (*Dicentrarchus labrax*) juveniles. *Aquaculture*.

[23] Lee S. M., Cho S. H., Kim K. D. (2000). Effects of dietary protein and energy levels on growth and body composition of juvenile flounder *Paralichthys olivaceus*. *Journal of the World Aquaculture Society*.

[24] Dong X. J., Qin W. H., Fu Y. Y. (2021). Effects of dietary betaine on cholesterol metabolism and hepatopancreas function in gibel carp (*Carassius gibelio*) fed with a high-fat diet. *Aquaculture Nutrition*.

[25] Liu K., Liu H., Chi S., Dong X., Tan B. (2018). Effects of different dietary lipid sources on growth performance, body composition and lipid metabolism-related enzymes and genes of juvenile golden pompano, *Trachinotus ovatus*. *Aquaculture Research*.

[26] Li X., Chen Q., Chen Q., Mai K., Ai Q. (2021). Effects of dietary terrestrial oils supplemented withl-carnitine on growth, antioxidant capacity, lipid metabolism and inflammation in large yellow croaker (*Larimichthys crocea*). *The British Journal of Nutrition*.

[27] Chen X., Niu J., Wang J., Zhao W. (2022). Effects of L-carnitine supplementation in high-fat diet on growth, antioxidant capacity and lipid metabolism of golden pompano (*Trachinotus ovatus*). *Frontiers in Marine Science*.

[28] Fang H. H., Niu J. (2022). Dietary supplementation of L-carnitine relieved detrimental impacts of a high-fat diet in juvenile *Trachinotus ovatus*. *Aquaculture Reports*.

[29] Froyland L., Madsen L., Eckhoff K. M. (1998). Carnitine palmitoyltransferase I, carnitine palmitoyltransferase II, and acyl-CoA oxidase activities in Atlantic salmon (Salmo salar). *Lipids*.

[30] Yang S. D., Liu F. G., Liou C. H. (2012). Effects of dietary L-carnitine, plant proteins and lipid levels on growth performance, body composition, blood traits and muscular carnitine status in juvenile silver perch (*Bidyanus bidyanus*). *Aquaculture*.

[31] Li Y. N., Pang Y. N., Xiang X. J., Du J. L., Mai K. S., Ai Q. H. (2019). Molecular cloning, characterization, and nutritional regulation of elovl 6 in large yellow croaker (*Larimichthys crocea*). *International Journal of Molecular Sciences*.

[32] Dolley G., Boisclair M. E., Lamarche B. (2011). Interactions between dietary fat intake and FASN genetic variation influence LDL peak particle diameter. *Journal of Nutrigenetics and Nutrigenomics*.

[33] Sanchez M., Lins-Rodrigues M., Pessini J. E., Bittencourt F., Boscolo W. R., Signor A. (2021). Dietary supplementation with l-carnitine for Nile tilapia juveniles. *Aquaculture*.

[34] Owen K. Q., Jit H., Maxwell C. V. (2001). Dietary L-carnitine suppresses mitochondrial branched-chain keto acid dehydrogenase activity and enhances protein accretion and carcass characteristics of swine. *Journal of Animal Science*.

[35] Chen Y., Sun Z., Liang Z. (2020). Effects of dietary fish oil replacement by soybean oil and L-carnitine supplementation on growth performance, fatty acid composition, lipid metabolism and liver health of juvenile largemouth bass, *Micropterus salmoides*. *Aquaculture*.

[36] Hossain M. A., Almatar S. M., James C. M. (2010). Optimum dietary protein level for juvenile silver pomfret, *Pampus argenteus* (Euphrasen). *Journal of the World Aquaculture Society*.

[37] Kim S. S., Lee K. J. (2009). Dietary protein requirement of juvenile tiger puffer (*Takifugu rubripes*). *Aquaculture*.

[38] Babatz T. D., Spear E. D., Xu W. (2021). Site specificity determinants for prelamin a cleavage by the zinc metalloprotease ZMPSTE24. *The Journal of Biological Chemistry*.

[39] Liu S., Young S. M., Varisco B. M. (2014). Dynamic expression of chymotrypsin-like elastase 1 over the course of murine lung development. *American Journal of Physiology-Lung Cellular and Molecular Physiology*.

[40] Bommer U. A., Iadevaia V., Chen J. Z., Knoch B., Engel M., Proud C. G. (2015). Growth-factor dependent expression of the translationally controlled tumour protein TCTP is regulated through the PI3-K/Akt/mTORC1 signalling pathway. *Cellular Signalling*.

[41] Sabatini D. M. (2017). Twenty-five years of mTOR: uncovering the link from nutrients to growth. *Proceedings of the National Academy of Sciences of the United States of America*.

