# Supplementary material for: Effect of Adding L-carnitine to High-Fat/Low-Protein Diets of Common Carp (Cyprinus carpio) and the Mechanism of Regulation of Fat and Protein Metabolism
Source: Aquac Nutr. 2022 Aug 23;2022:3768368. doi: 10.1155/2022/3768368 (PMC9980285; doi:10.1155/2022/3768368)
Supplement: Supplementary 1 — Supplementary Table 1: real-time PCR primer sequences. [file 3768368.f1.docx]

| Table S1 Real-time PCR primer sequences | | |
| --- | --- | --- |
| Genes | Forward primer(5'-3') | Reverse primer(3'-5') |
| 1elovl6 | GAAGTCATCAAAATGGAATGGGA | TGCTTTCGGTACAGTTACCT |
| 2lss | TCCATTGACATGGCTTTCGT | AAAGGACACACCTGTGACCC |
| 3mtor | TCCCACATATGCTGCGAGTC | CGGGCGTTAAATCGTCTCTG |
| 4gatm | GTACGATCAGGTGACGCACA | TCAAGCTCGGCACAAAGCTA |
| β-actin | TACCACTTCGCCCTCATCAC | TGTCAAACAACGTTAATGCCCC |
| 1. elovl6, elongation of long-chain fatty acid family member 6; 2. lss, lanosterol synthase; 3. mtor,mammalian target of rapamycin; 4. gatm, glycine amidinotransferase. | | |
|  |  |  |
|  |  |  |
