# Supplementary material for: Effect of Adding L-carnitine to High-Fat/Low-Protein Diets of Common Carp (Cyprinus carpio) and the Mechanism of Regulation of Fat and Protein Metabolism
Source: Aquac Nutr. 2022 Aug 23;2022:3768368. doi: 10.1155/2022/3768368 (PMC9980285; doi:10.1155/2022/3768368)
Supplement: Supplementary 2 — Supplementary Table 2: clean data obtained from RNA sequencing. [file 3768368.f2.docx]

| Table S2 Clean data obtained from RNA sequencing | | | | | | |
| --- | --- | --- | --- | --- | --- | --- |
| Sample name | Reads number | Clean reads | Total mapped | Mapping rate(%) | Q20（%） | Q30（%） |
| A1 | 39078902 | 36338152 | 28104935 | 77.34 | 97.18 | 93.34 |
| A2 | 43565862 | 40560506 | 31341544 | 77.27 | 97.25 | 93.46 |
| A3 | 42850366 | 39913760 | 30835747 | 77.26 | 97.14 | 93.3 |
| B1 | 38594610 | 35874516 | 28099995 | 78.33 | 97.2 | 93.4 |
| B2 | 41861388 | 38955232 | 30766201 | 78.98 | 97.5 | 94.02 |
| B3 | 39028900 | 36370724 | 28647473 | 78.77 | 97.25 | 93.51 |
| C1 | 44439570 | 41360862 | 33013362 | 79.82 | 97.77 | 94.63 |
| C2 | 47874146 | 44591914 | 35552043 | 79.73 | 97.73 | 94.55 |
| C3 | 42903736 | 39972864 | 31865121 | 79.72 | 97.76 | 94.61 |
| Note: A. Diet 1; B. Diet 2; C. Diet 3. Same below | | | | | | |
