# Supplementary material for: Effect of Adding L-carnitine to High-Fat/Low-Protein Diets of Common Carp (Cyprinus carpio) and the Mechanism of Regulation of Fat and Protein Metabolism
Source: Aquac Nutr. 2022 Aug 23;2022:3768368. doi: 10.1155/2022/3768368 (PMC9980285; doi:10.1155/2022/3768368)
Supplement: Supplementary 3 — Supplementary Table 3: top 20 KGEE items with significant enrichment in Diet 1 vs. Diet 2. [file 3768368.f3.docx]

| Table S3 Top 20 KGEE items with significant enrichment in Diet 1 vs Diet 2 | | | | |
| --- | --- | --- | --- | --- |
| Pathway | Up-regulated | Down-regulated | Total numbe | P-value |
| Steroid biosynthesis | 2 | 12 | 34 | 1.50E-09 |
| Ribosome biogenesis in eukaryotes | 1 | 27 | 139 | 3.40E-09 |
| PPAR signaling pathway | 22 | 4 | 172 | 4.20E-06 |
| Metabolism of xenobiotics by cytochrome P450 | 5 | 9 | 77 | 9.10E-05 |
| alpha-Linolenic acid metabolism | 9 | 2 | 58 | 3.40E-04 |
| ECM-receptor interaction | 7 | 19 | 243 | 1.30E-03 |
| FoxO signaling pathway | 25 | 13 | 403 | 1.40E-03 |
| Linoleic acid metabolism | 7 | 2 | 49 | 1.50E-03 |
| Ferroptosis | 7 | 8 | 114 | 1.90E-03 |
| Glutathione metabolism | 5 | 10 | 116 | 2.20E-03 |
| NOD-like receptor signaling pathway | 22 | 14 | 389 | 2.50E-03 |
| Apoptosis | 24 | 11 | 380 | 3.10E-03 |
| Adipocytokine signaling pathway | 18 | 4 | 210 | 3.90E-03 |
| Fatty acid biosynthesis | 6 | 1 | 37 | 4.10E-03 |
| Glycerophospholipid metabolism | 18 | 8 | 270 | 5.60E-03 |
| Drug metabolism - cytochrome P450 | 5 | 6 | 81 | 5.60E-03 |
| Drug metabolism - other enzymes | 8 | 7 | 133 | 8.00E-03 |
| Steroid hormone biosynthesis | 5 | 6 | 87 | 9.60E-03 |
| Riboflavin metabolism | 3 | 1 | 16 | 0.0106 |
