# Supplementary material for: Effect of Adding L-carnitine to High-Fat/Low-Protein Diets of Common Carp (Cyprinus carpio) and the Mechanism of Regulation of Fat and Protein Metabolism
Source: Aquac Nutr. 2022 Aug 23;2022:3768368. doi: 10.1155/2022/3768368 (PMC9980285; doi:10.1155/2022/3768368)
Supplement: Supplementary 4 — Supplementary Table 4: differential genes related to fat metabolism or protein metabolism in Diet 1 vs. Diet 2. [file 3768368.f4.docx]

| Table S4 Differential genes related to fat metabolism or protein metabolism in Diet 1 vs Diet 2 | | | | |
| --- | --- | --- | --- | --- |
| Gene | Metabolism | Level | Log2FC1 | P-value |
| *elovl6* | Lipid | Up-regulation | 2.875 | 3.01E-65 |
| *lss* |  | Down-regulation | -1.828 | 2.59E-15 |
| *ptgs2* |  | UP-regulation | 1.105 | 0.0216 |
| *kdsr* |  | UP-regulation | 1.41 | 0.0845 |
| *lipc* |  | Down-regulation | -1.278 | 7.41E-54 |
| *hmgcs1* |  | Down-regulation | -1.495 | 3.24E-35 |
| *phgdh* | Protein | Up-regulation | 1.09 | 5.55E-10 |
| *gatm* |  | Down-regulation | -1.14 | 1.08E-22 |
| *dmgdh* |  | Down-regulation | -1.362 | 5.20E-35 |
| *gss* |  | Down-regulation | -1.785 | 2.27E-32 |
| *srm* |  | Down-regulation | -1.027 | 0.0191 |
| *sat2* |  | Down-regulation | -1.624 | 0.0179 |
| *hmgcs1* |  | Down-regulation | -1.495 | 3.24E-35 |
| *fah* |  | Down-regulation | -1.895 | 8.86E-38 |
| Note: 1 Log2FC: log2Foldchange (Diet 1 vs Diet 2). | | | | |
