# Supplementary material for: Effect of Adding L-carnitine to High-Fat/Low-Protein Diets of Common Carp (Cyprinus carpio) and the Mechanism of Regulation of Fat and Protein Metabolism
Source: Aquac Nutr. 2022 Aug 23;2022:3768368. doi: 10.1155/2022/3768368 (PMC9980285; doi:10.1155/2022/3768368)
Supplement: Supplementary 5 — Supplementary Table 5: top 20 KEGG items with significant enrichment in Diet 2 vs. Diet 3. [file 3768368.f5.docx]

| Table S5 Top 20 KEGG items with significant enrichment in Diet 2 vs Diet 3 | | | | |
| --- | --- | --- | --- | --- |
| Pathway | Up-regulated | Down-regulated | Total numbe | P-value |
| Steroid biosynthesis | 14 | 5 | 34 | 5.60E-12 |
| PPAR signaling pathway | 10 | 29 | 172 | 4.60E-08 |
| Fatty acid biosynthesis | 0 | 14 | 37 | 1.70E-06 |
| Glycolysis / Gluconeogenesis | 12 | 14 | 121 | 2.10E-05 |
| ABC transporters | 9 | 13 | 94 | 2.20E-05 |
| Fructose and mannose metabolism | 12 | 8 | 85 | 4.70E-05 |
| Ferroptosis | 13 | 11 | 114 | 6.00E-05 |
| Linoleic acid metabolism | 2 | 12 | 49 | 6.70E-05 |
| Glycerophospholipid metabolism | 7 | 37 | 270 | 7.30E-05 |
| Arginine and proline metabolism | 8 | 12 | 93 | 1.70E-04 |
| Pyruvate metabolism | 3 | 14 | 73 | 1.90E-04 |
| Riboflavin metabolism | 2 | 5 | 16 | 2.50E-04 |
| Cysteine and methionine metabolism | 5 | 13 | 83 | 3.30E-04 |
| Metabolism of xenobiotics by cytochrome P450 | 4 | 13 | 77 | 3.80E-04 |
| alpha-Linolenic acid metabolism | 2 | 12 | 58 | 4.70E-04 |
| Steroid hormone biosynthesis | 4 | 14 | 87 | 6.00E-04 |
| Glycine, serine and threonine metabolism | 3 | 12 | 68 | 8.30E-04 |
| Pentose phosphate pathway | 7 | 6 | 55 | 9.10E-04 |
| Insulin signaling pathway | 25 | 30 | 401 | 9.90E-04 |
| One carbon pool by folate | 2 | 7 | 31 | 1.20E-03 |
