# Supplementary material for: Effect of Adding L-carnitine to High-Fat/Low-Protein Diets of Common Carp (Cyprinus carpio) and the Mechanism of Regulation of Fat and Protein Metabolism
Source: Aquac Nutr. 2022 Aug 23;2022:3768368. doi: 10.1155/2022/3768368 (PMC9980285; doi:10.1155/2022/3768368)
Supplement: Supplementary 6 — Supplementary Table 6: differential genes related to fat metabolism or protein metabolism in Diet 2 vs. Diet 3. [file 3768368.f6.docx]

| Table S6 Differential genes related to fat metabolism or protein metabolism in Diet 2 vs Diet 3 | | | | |
| --- | --- | --- | --- | --- |
| Gene | Metabolism | Level | Log2FC1 | P-value |
| *lss* | Lipid | Up-regulation | 2.212 | 4.11E-26 |
| *gpam* |  | Down-regulation | -1.124 | 6.40E-40 |
| *hsd11b2* |  | Down-regulation | -1.063 | 9.10E-06 |
| *ptgs2* |  | Up-regulation | 1.227 | 5.30E-06 |
| *pla2g12b* |  | Down-regulation | -1.197 | 3.80E-58 |
| *tkfc* |  | Down-regulation | -1.428 | 1.10E-46 |
| *elovl6* |  | Down-regulation | -2.977 | 5.00E-194 |
| *hmgcs1* |  | Up-regulation | 1.006 | 1.30E-16 |
| *cpt1a* |  | Up-regulation | 2.043 | 3.10E-83 |
| *cpt2* |  | Up-regulation | 1.346 | 0.0349 |
| fasn |  | Down-regulation | -1.933 | 5.40E-40 |
| *srm* | Protein | Up-regulation | 1.095 | 0.0335 |
| *got1* |  | Down-regulation | -1.487 | 1.80E-21 |
| *cth* |  | Down-regulation | -1.036 | 3.10E-09 |
| *mtor* |  | up-regulation | 1.274 | 8.20E-29 |
| *gatm* |  | Up-regulation | 1.668 | 1.60E-55 |
| *gcat* |  | Down-regulation | -2.05 | 0.0449 |
| *psph* |  | Down-regulation | -2.493 | 2.10E-20 |
| *pipox* |  | Down-regulation | -1.488 | 2.70E-32 |
| *hmgcs1* |  | Up-regulation | 1.006 | 1.30E-16 |
| *asns* |  | Down-regulation | -1.211 | 3.90E-07 |
| *acmsd* |  | Down-regulation | -1.103 | 0.0216 |
| *dot1l* |  | Up-regulation | 1.695 | 0.0041 |
| *bbox1* |  | Up-regulation | 2.257 | 2.00E-54 |
| *hal* |  | Down-regulation | -1.698 | 7.40E-36 |
| *uroc1* |  | Down-regulation | -1.835 | 2.00E-145 |
| *sae1* |  | Up-regulation | 1.196 | 0.0013 |
| *ube2c* |  | Up-regulation | 2.849 | 0.0108 |
| Note: 1 Log2FC: log2Foldchange (Diet 2 vs Diet 3). | | | | |
