# Supplementary material for: Effect of Adding L-carnitine to High-Fat/Low-Protein Diets of Common Carp (Cyprinus carpio) and the Mechanism of Regulation of Fat and Protein Metabolism
Source: Aquac Nutr. 2022 Aug 23;2022:3768368. doi: 10.1155/2022/3768368 (PMC9980285; doi:10.1155/2022/3768368)
Supplement: Supplementary 7 — Supplementary Table 7: differentially expressed genes related to fat or protein metabolism. [file 3768368.f7.docx]

| Table S7 Differentially expressed genes related to fat or protein metabolism | | | | | |
| --- | --- | --- | --- | --- | --- |
| Gene name |  | Participate in metabolic process | Relative expression 1 | Log2(foldchange) | Q-valuei |
| *elovl6* | A vs B | Fatty acid elongation | Up | 2.875 | 3.01E-65 |
|  | A vs C |  | Down | -1.455 | 7.22E-39 |
|  | B vs C |  | Down | -2.977 | 4.90E-194 |
| *lipc* | A vs B | Lipoprotein metabolism | Down | -1.278 | 7.41E-54 |
|  | A vs C |  | Down | -1.987 | 1.38E-168 |
| *pik3r3* | A vs B | Regulation of phosphatidylinositol 3 kinase activity | Up | 1.561 | 0.0136 |
|  | A vs C |  | Up | 1.99 | 3.97E-05 |
| *cpt1a* | A vs C | Fatty acid catabolism | Up | 1.7 | 4.87E-80 |
|  | B vs C |  | Up | 2.043 | 3.10E-83 |
| *cpt2* | B vs C | Fatty acid catabolism | Up | 1.346 | 0.0349 |
| *fasn* | A vs C | Fatty acid synthesis | Down | -1.007 | 3.80E-05 |
|  | B vs C |  | Down | -1.933 | 5.40E-40 |
| *acmsd* | A vs C | Cellular biogenic amine metabolism | Down | -1.222 | 6.87E-09 |
|  | B vs C |  | Down | -1.103 | 0.0216 |
| *sars2* | A vs C | Serinyl tRNA aminoacylation | Down | -1.056 | 0.03 |
| *wars* | A vs C | Ryptophanyl tRNA aminoacylation | Down | -1.607 | 1.25E-16 |
|  | B vs C |  | Down | -1.45 | 2.81E-13 |
| *lars* | A vs B | Aminoacylation of tRNA in protein translation | Down | -1.239 | 1.01E-11 |
| *gss* | A vs B | Glutathione metabolism | Down | -1.785 | 2.27E-32 |
|  | A vs C |  | Down | -1.379 | 2.96E-25 |
| *mtor* | A vs B | Protein synthesis process | Down | -1.274 | 8.17E-29 |
|  | B vs C |  | Up | 1.677 | 0.1518 |
| *gch1* | A vs B | Tetrahydrofolate biosynthesis process | Down | -1.386 | 7.67E-10 |
|  | A vs C |  | Down | -1.09 | 6.46E-12 |
| *pop5* | A vs C | Amino acid modification | Down | -1.209 | 0.0051 |
| *cela1* | A vs C | Proteolysis | Down | -5.296 | 0 |
|  | B vs C |  | Down | -4.391 | 7.93E-96 |
| *cpb1* | A vs C | Proteolysis | Down | -4.702 | 0 |
|  | B vs C |  | Down | -3.709 | 3.94E-105 |
| *zmpste24* | A vs B | Proteolysis | Down | -1.377 | 1.15E-13 |
|  | B vs C |  | Up | -3.709 | 3.90E-105 |
| *tmem27* | A vs B | Proteolysis | Down | -2.986 | 0.0036 |
|  | A vs C |  | Down | -3.007 | 0.0015 |
| *pm20d1* | A vs B | Proteolysis | Down | -1.179 | 0.0158 |
| *gatm* | B vs C | α-amino acid biosynthesis process | Up | 1.668 | 1.62E-55 |
| Note: 1 Take one group before "vs" as the reference, and the other group as the expression amount of the gene. Same below. | | | | | |
